# Efficacy and safety of immune checkpoint inhibitor rechallenge therapy in the treatment of advanced acquired immune-resistant non-small cell lung cancer: a retrospective study

**DOI:** 10.3389/fonc.2025.1621860

**Published:** 2025-09-22

**Authors:** Jian Wang, Qijia Gao, Jianxin Chen

**Affiliations:** ^1^ Department of Education, International Word, The Quzhou Affiliated Hospital of Wenzhou Medical University, Quzhou People′s Hospital, Quzhou, Zhejiang, China; ^2^ Department of Gastroenterology, Jiaxing Second Hospital, Jiaxing, Zhejiang, China; ^3^ Changsha Medical University, Changsha, Hunan, China

**Keywords:** non-small cell lung cancer, immune checkpoint inhibitor, rechallenge, efficacy, adverse events

## Abstract

**Background:**

Patients with advanced non-small cell lung cancer who have failed first-line immunotherapy and lack driver gene mutations face limited options for subsequent treatment. A working group recently proposed updated clinical diagnostic criteria for acquired immune resistance. Based on these criteria, this study evaluated the efficacy and safety of immune rechallenge therapy in patients with advanced NSCLC exhibiting acquired resistance.

**Methods:**

The study involved 13 patients diagnosed with advanced immune-acquired resistance NSCLC. These patients initially exhibited a partial response to immunotherapy but experienced disease progression within six months following their last immune checkpoint inhibitors (ICIs) treatment. Subsequently, they received ICIs again. The outcomes assessed included the objective response rate (ORR), disease control rate (DCR), progression-free survival (PFS), overall survival (OS), and safety. PFS1 refers to the time from the first administration of anti–PD-1/PD-L1 blockade therapy to PD. PFSR was defined as the duration from the first day of the second ICIs administration to disease progression, death, or the last follow-up date. OS was defined as the time from the first day of the second ICIs administration to the date of death or the last follow-up date.

**Results:**

The median age was 67 years, and 76.9% of patients were male. The disease control rate (DCR) was 61.5%, with an ORR of 0%. The median PFSR was 2.90 months (95% CI, 1.97–3.83), and the median PFS1 was 5.97 months (95% CI, 4.13–7.81). Poor ECOG performance status was significantly associated with shorter PFS (HR = 6.839, 95% CI: 1.557–30.032, p = 0.011).During initial ICIs treatment, the most common adverse events were fatigue (46.1%) and anemia (38.5%). Grade 3–4 toxicities included anemia and neutropenia (15.4% each), leukopenia (7.8%), and fatigue (7.8%). In the ICIs rechallenge phase, anemia (38.5%) and fatigue (30.7%) remained the most frequent adverse events, with only one Grade 3–4 event reported (anemia, 7.8%).

**Conclusions:**

Patients with advanced non-small cell lung cancer who exhibit immune-acquired resistance may still derive clinical benefit from rechallenging with immune checkpoint inhibitors, particularly in those with a favorable ECOG performance status. Further prospective clinical trials and molecular investigations are necessary to validate these findings and better define the patient subgroups most likely to benefit from this therapeutic approach.

## 1 Introduction

Lung cancer, a significant global public health burden, is currently the leading cause of cancer-related deaths (1). In 2025, the American Cancer Society estimates that there will be 226650 new cases and 124730 lung cancer-related deaths in the United States (2). Non-small cell lung cancer (NSCLC) is the most prevalent type, representing approximately 85% of all lung cancer cases, with more than half of NSCLC cases diagnosed at an advanced stage (3). Currently, the ASCO guidelines classify the first-line treatment of patients with advanced NSCLC into two primary approaches: targeted therapy and immunotherapy (IO), which are determined by the presence of driver alterations. For advanced NSCLC patients without targetable oncogenes, the guidance recommends monotherapy with immune checkpoint inhibitors (ICIs) or their combination with chemotherapy as the first-line treatment, achieving a five-year survival rate of 20% to 30% (4).

Although ICI-based therapies have become the standard first-line option for these patients, less than 50% exhibit a response to initial IO (5, 6), and 65% of those who initially respond will experience disease progression (PD) due to IO resistance within four years (7, 8). Follow-up treatment options for these patients are significantly limited, and there is a notable lack of optimal treatment strategies. At present, the predominant approach focuses on extending these patients’ survival through various combination therapies, which may include other immunotherapeutic agents, chemotherapy, anti-angiogenic drugs, and radiotherapy (9). Among these, immune rechallenge therapy has garnered attention from numerous experts and scholars as a viable and promising treatment strategy (10). However, before considering the rechallenge of ICIs in advanced NSCLS patients, it is crucial to distinguish between primary immune resistance (PIR) and acquired immune resistance (AIR), as the mechanisms underlying these forms of resistance differ (11). These mechanisms include alterations in β2-microglobulin, interferon-γ (IFN-γ) signaling, neoantigen loss, tumor-mediated immunosuppression/exclusion, and additional inhibitory checkpoints (12). PIR refers to the failure to respond to initial IO, leading to PD, while AIR indicates an initial response to IO followed by subsequent PD (7). Notably, a significant challenge in lung cancer IO is the absence of a unified standard for AIR. Furthermore, the incidence of AIR in lung cancer is notably higher than in other tumors (7). To address this clinical challenge, Schoenfeld et al. (12) proposed a definition of AIR that aligns more closely with clinical practice for advanced NSCLC patients, as follows: 1. Prior treatment with IO; 2. Objective response to PD(L)-1 blockade (stable disease (SD) is excluded); and 3. Progression occurring within 6 months of the last PD(L)-1 blockade therapy (**Table 1**). To identify patients suitable for ICIs rechallenge therapy, we adopted this new criterion of immune resistance and conducted a retrospective analysis of the relationship between the clinical characteristics of NSCLC patients and the efficacy of ICIs rechallenge.

**Table 1:** Definition of acquired immune resistance to PD-(L)1 blockade in advanced NSCLC.

Category	Criteria
Treatment exposure	Patients must have received PD-(L)1 blockade therapy.
Initial response	An objective response is defined as a complete response or partial response; Stable disease is excluded.
Progression timing	Disease progression occurs within 6 months after the last PD-(L)1 treatment.

## 2 Methods

### 2.1 Patients

This study retrospectively analyzed the data of patients with advanced NSCLC who were diagnosed with AIR and received immune checkpoint inhibitor (ICI) rechallenge treatment at Quzhou People’s Hospital between May 2020 and December 1, 2024. Data and follow-up records were last updated on December 1, 2024. The inclusion criteria were as follows: 1. Patients with histologically or cytologically confirmed unresectable advanced (stage III or IV) or recurrent NSCLC; 2. Patients who met the diagnostic criteria for AIR, as shown in **Table 1**; **2**. All included patients had at least one measurable lesion; 4. Patients aged between 18 to 75 years. The exclusion criteria included: 1. Patients who discontinued initial ICI-based therapies due to clinical decisions, bone marrow suppression, or immune-related adverse events (irAEs); 2. Incomplete clinical data and lack of data to evaluate treatment effects; 3. A poor Eastern Cooperative Oncology Group (ECOG) performance status (PS) of >2. This study was approved by the ethical committee of Quzhou People’s Hospital. All investigations were conducted following the declaration of Helsinki (revised in 2013).

**Table 2:** Efficacy of ICIs rechallenge therapy in advanced NSCLC patients with acquired immune resistance (n = 13).

Efficacy	All patients (n = 13)
Complete response (%)	0
Partial response (%)	0
Stable disease (%)	8 (61.5)
Progressive disease (%)	5 (38.5)
Objective response rate (%, CR, PR)	0
Disease control rate (%, CR, PR, SD)	8 (61.5)
median progression-free survival (months, 95% CI)	2.90 (1.97, 3.83)

### 2.2 General information collection

The baseline characteristics and tumor treatment data of patients who met the specified criteria were collected from the hospital’s electronic medical record system. This data included age, sex, smoking status, ECOG PS, tumor TNM stage, histological subtype, PD-L1 expression, initial immunotherapy regimen, and ICIs rechallenge regimen, etc.

### 2.3 Outcomes and efficacy evaluations

Efficacy endpoints included progression-free survival (PFS), defined as the time from the initiation of study treatment to PD or death from any cause; overall survival (OS), defined as the time from the first day of the second ICIs administration to the date of death or the last follow-up date; overall response rate (ORR), defined as the proportion of patients achieving a complete response (CR) or partial response (PR); and disease control rate (DCR), defined as the proportion of patients with CR, PR, or SD according to the Response Evaluation Criteria in Solid Tumors (RECIST) version 1.1. The PFS for ICI-based therapy (PFS1) refers to the time from the first administration of anti–PD-1/PD-L1 blockade therapy to PD, while the PFS for ICIs rechallenge regimens (PFSR) refers to the time from immune rechallenge to PD. Patients without documented clinical or radiographic disease progression, or those who remained alive, were censored at the last follow-up date. Adverse events (AEs) were graded according to the National Cancer Institute Common Terminology Criteria for Adverse Events version 4.0 (NCI-CTCAE v4.0).

### 2.4 Statistical analysis

Continuous data are presented as means and standard deviations, or as medians and interquartile ranges (IQR), and as frequencies and percentages for categorical variables. Survival curves were calculated using the Kaplan–Meier method and compared using the log-rank test based on ECOG PS and PD-L1 status. Kaplan–Meier survival curves were generated using R software (v4.4.1) with the survival and survminer packages. Subgroup survival analyses were also performed according to ECOG performance status and PD-L1 expression levels. Hazard ratios (HRs) and their 95% confidence intervals (CIs) were estimated using the Cox proportional hazards model. P < 0.05 was considered statistically significant.

## 3 Results

### 3.1 Patient characteristics

A total of 13 patients with advanced NSCLC lacking targetable gene mutations and diagnosed with AIR underwent immune rechallenge therapy. The median age of the cohort was 67 years (range: 58-70), with 10 patients (76.9%) being male and 10 patients (76.9%) having a history of smoking. According to the 8th edition of the TNM classification for lung cancer, 2 patients (15.4%) were classified as stage III, while 11 patients (84.6%) were classified as stage IV. Histological analysis revealed that 6 patients (46.1%) had squamous cell carcinoma, 6 patients (46.1%) had adenocarcinoma, and 1 patient (7.8%) had adenosquamous carcinoma. Among the patients, 6 (46.1%) had lung metastases, 2 (15.4%) had brain metastases, and 2 (15.4%) had bone metastases. Additionally, 5 patients (38.5%) exhibited negative PD-L1 expression, and 5 patients (38.5%) had an ECOG PS of 2. Most patients received interim treatments between initial and rechallenge ICIs therapy. **Table 3**, **Appendix 1** and **Appendix 2** present the characteristics of the patients.

**Table 3:** Baseline characteristics of the 13 patients with advanced NSCLC.

Baseline characteristics	All patients (n = 13)
Age (years), n (%)	
Median (range)	67 (58-70)
≥60	8 (61.5)
<60	5 (38.5)
Gender, n (%)	
Male	10 (76.9)
Female	3 (23.1)
TNM stage, n (%)	
III	2 (15.4)
IV	11 (84.6)
Smoking status, n (%)	
Nonsmoker	3 (23.1)
Former smoker/smoker	10 (76.9)
Histological category	
Adenocarcinoma	6 (46.1)
Squamous cell carcinoma	6 (46.1)
Adenosquamous carcinoma	1 (7.8)
Presence of metastases, n (%)	
Brain	2 (15.4)
Lung	6 (46.1)
Bone	2 (15.4)
No metastasis	3 (23.1)
BMI index, n (%)	
< 18.5	3 (23.1)
18.5–23.9	6 (46.1)
≥ 24.0	4 (30.8)
Number of metastatic tumors, n (%)	
< 5	9 (69.1)
5–10	3 (23.1)
≥ 10	1 (7.8)
PD-L1 expression, n (%)	
< 1%	5 (38.5)
1%–50%	7 (53.7)
≥ 50%	1 (7.8)
ECOG PS, n (%)	
0–1	8 (61.5)
2	5 (38.5)
Interim treatments between initial and rechallenge ICI therapy, n (%)	
Yes	10 (76.9)
No	3 (23.1)
PFS1 time (months), n (%)	
<6	7 (53.9)
≥6	6 (46.1)

### 3.2 Efficacy and safety of ICIs treatments

**Figure 1:**
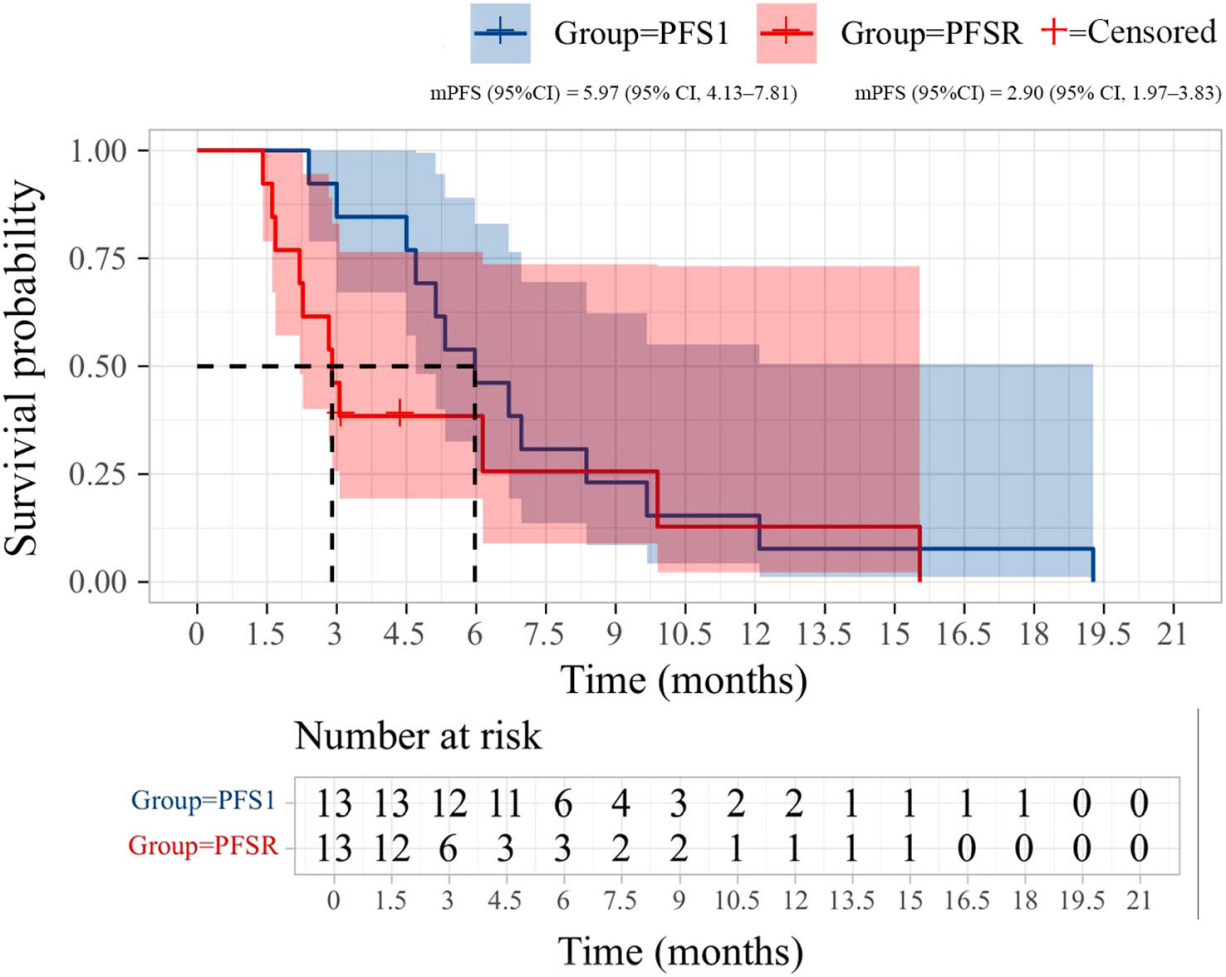
Kaplan-Meier curves of progression-free survival (PFS) during initial immunotherapy and immunotherapy rechallenge in 13 patients.

**Figure 2:**
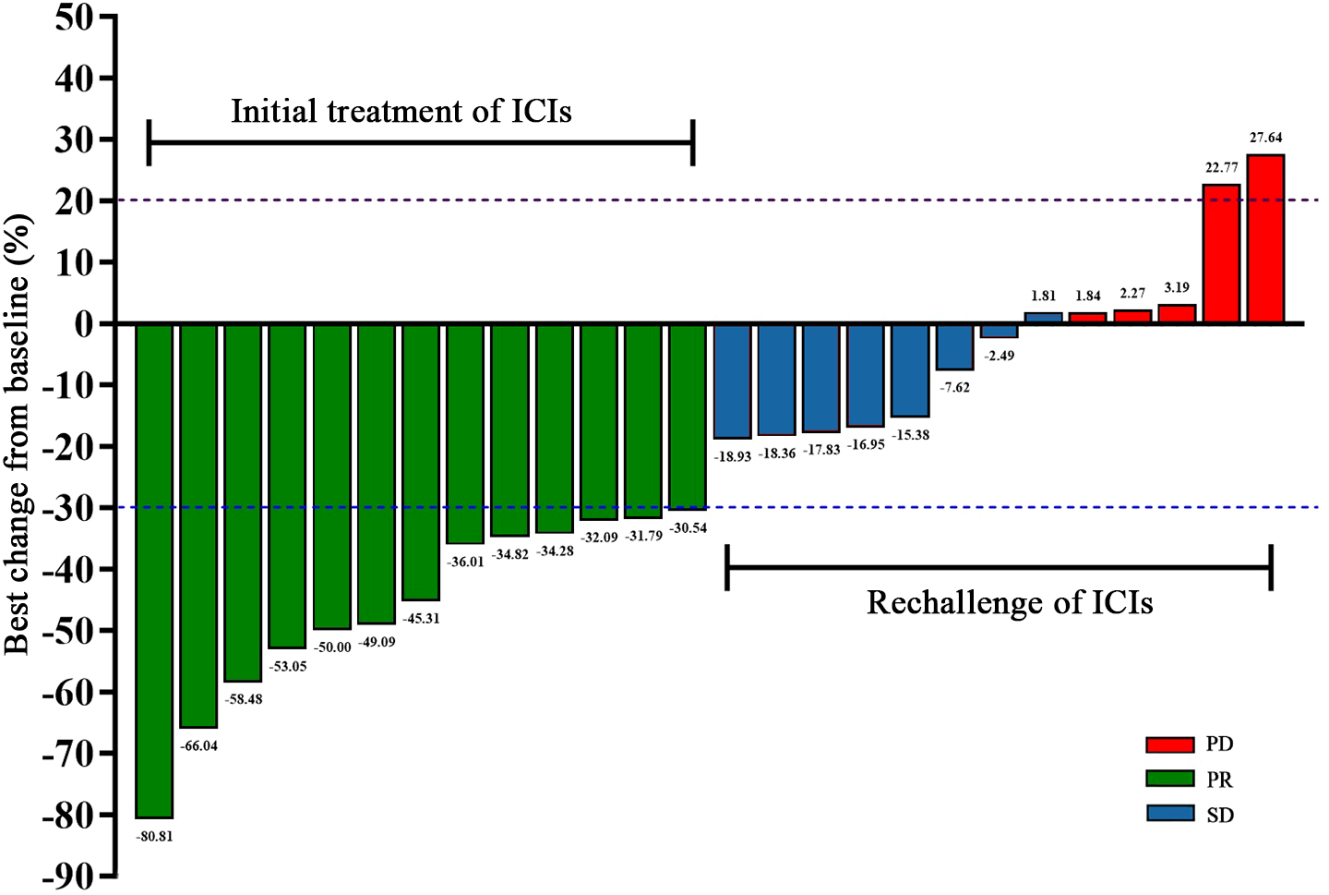
Waterfall plot of initial immunotherapy and immunotherapy rechallenge in 13 patients.

**Figure 3:**
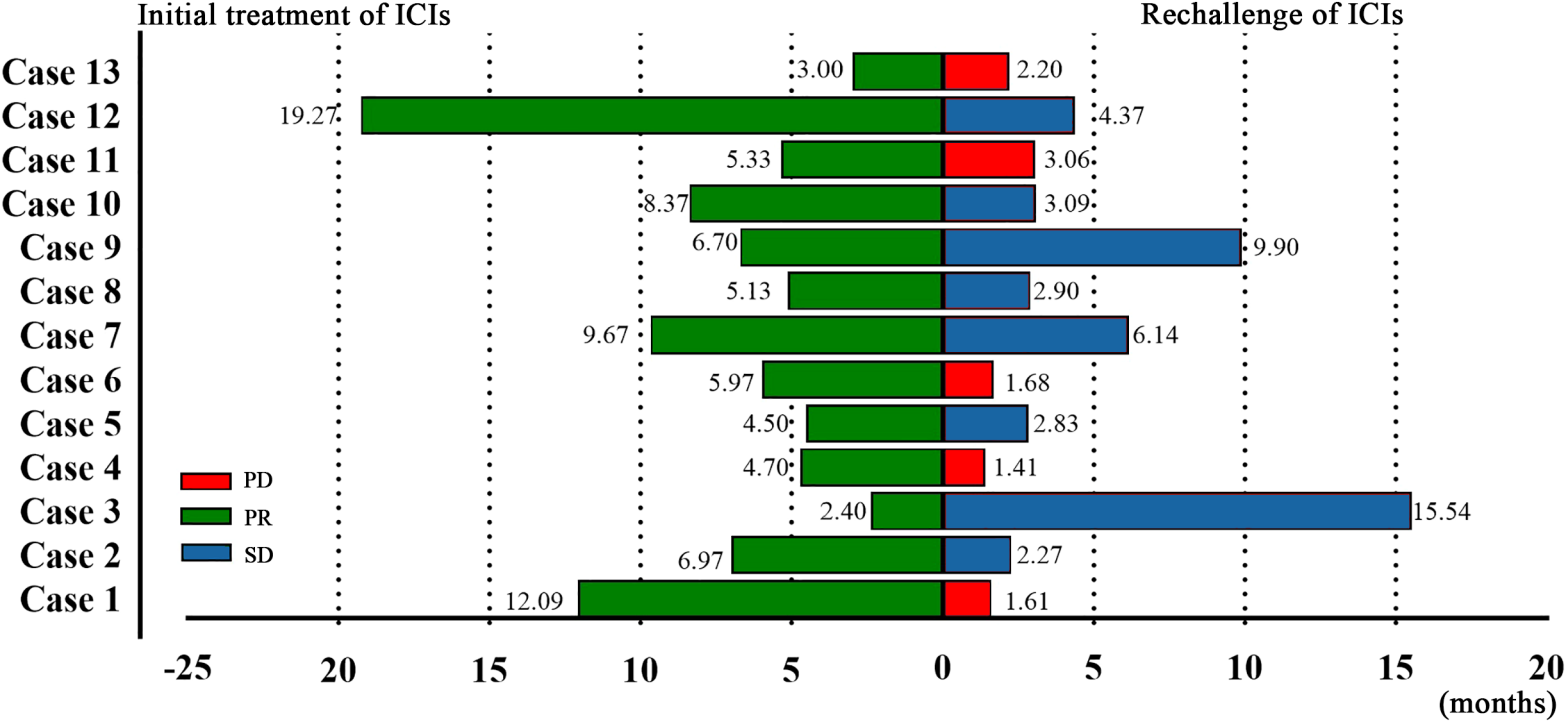
Swimmer plot of initial immunotherapy and immunotherapy rechallenge in 13 patients.

After the initial ICIs treatment, all patients achieved PR, with a median PFS of 5.97 months (95% CI, 4.13–7.81; **Figure 1**). Following the rechallenge of ICIs, 8 patients (61.5%) achieved SD, while 5 patients (38.5%) experienced PD. The DCR was 61.5%, while the ORR was 0% (**Table 2**). The median PFS was 2.90 months (95% CI, 1.97-3.83; **Figure 1**). The overall response of target lesions in the entire cohort following both the initial ICIs treatment and the rechallenge of ICIs is illustrated in **Figure 2**, and the PFS results are presented in **Figure 3**. OS data were immature.

### 3.3 Subgroup analysis and prognostic factors for PFSR

**Figure 4:**
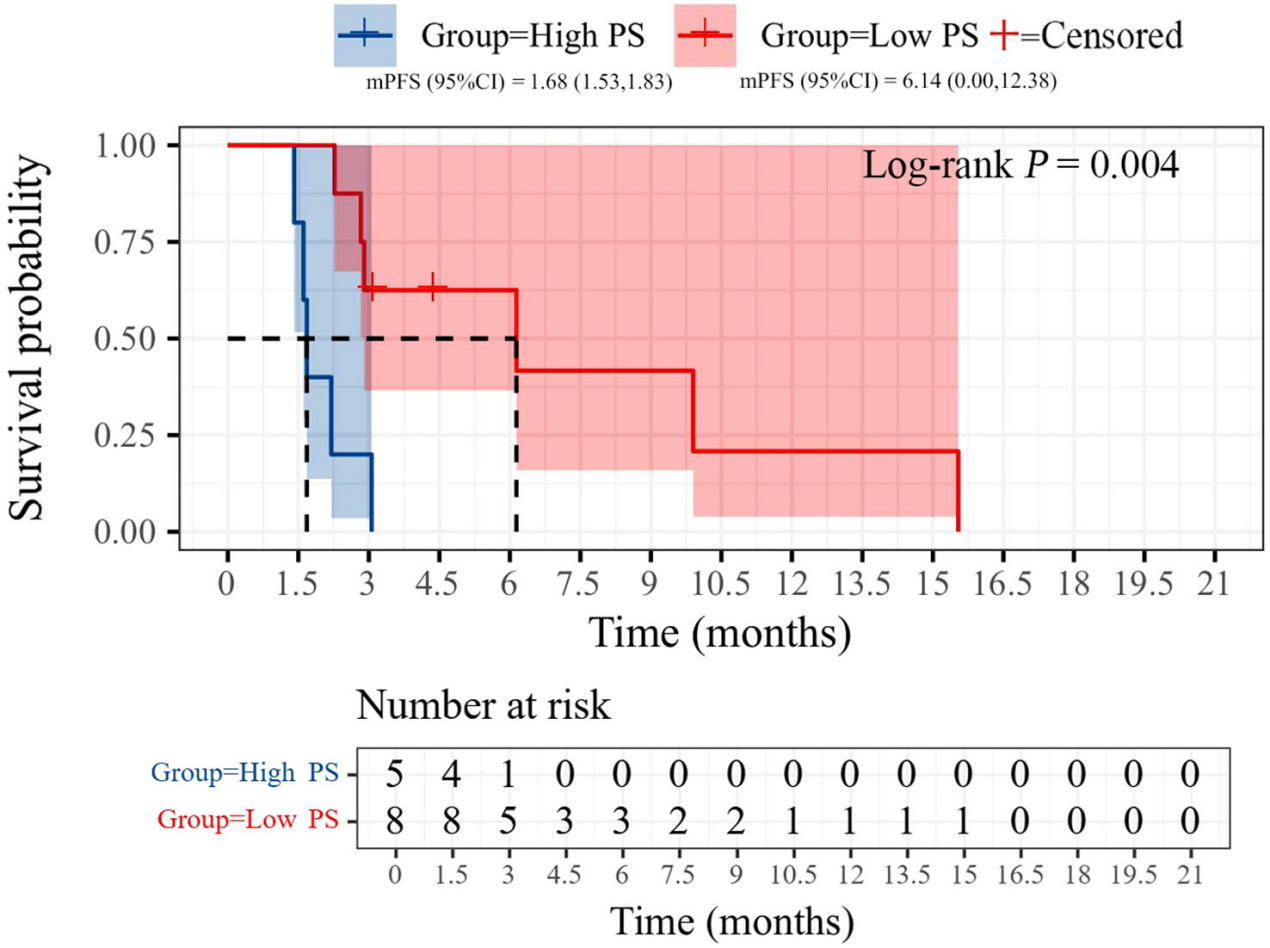
Kaplan-Meier curves of PFS during immunotherapy rechallenge by PD-1 expression status.

**Figure 5:**
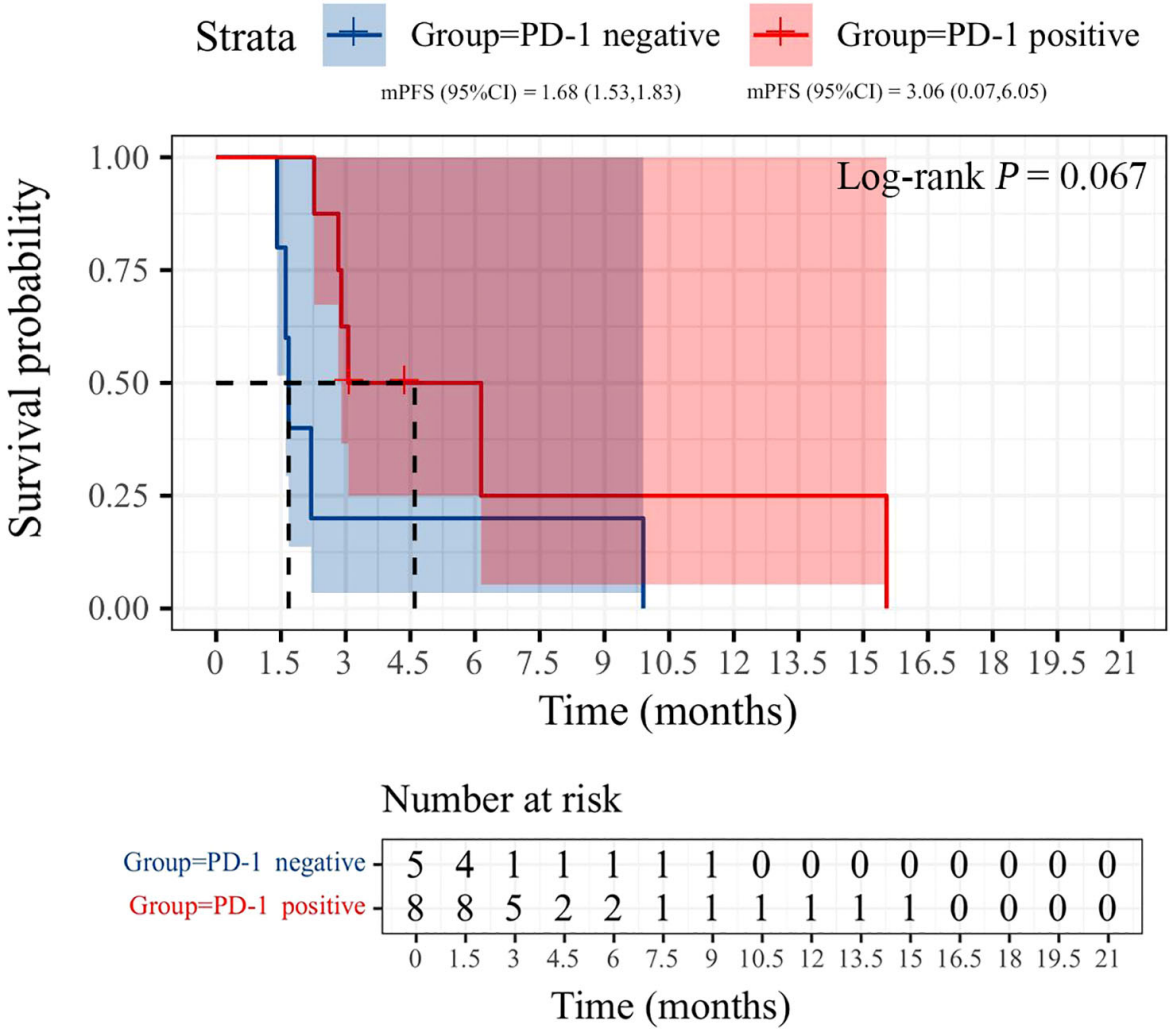
Kaplan-Meier curves of PFS during immunotherapy rechallenge by ECOG PS.

**Figure 6:**
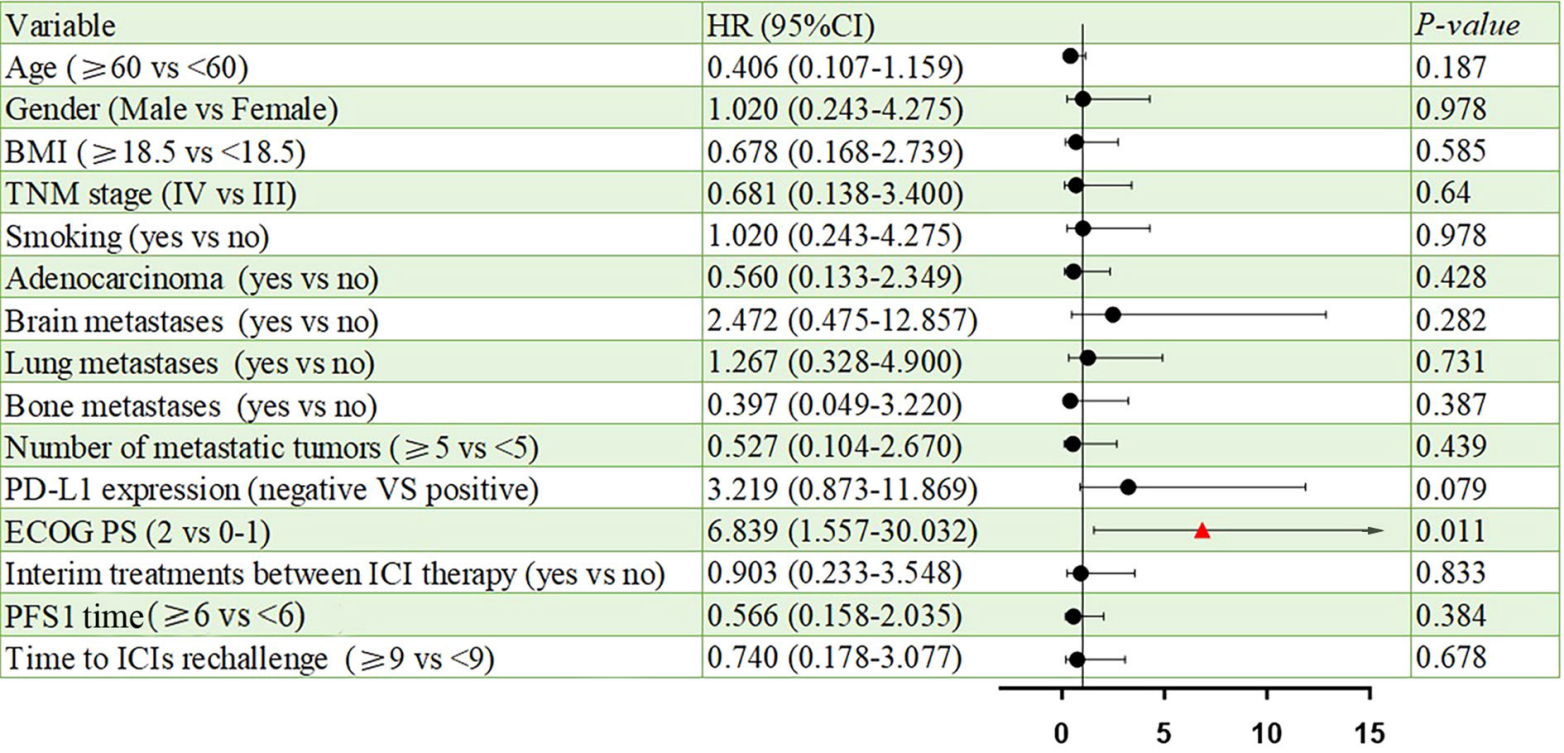
Univariate analysis of prognostic factors for PFS.

All patients were categorized into two subgroups based on PD-L1 status and PS score. As illustrated in **Figure 4**, the PFS of the low PS group was significantly longer than that of the high PS group, with durations of 6.14 months and 1.68 months, respectively (p = 0.004). In contrast, no significant difference in PFS was observed between the two groups when stratified by PD-L1 status (**Figure 5**). Furthermore, univariate analysis of patient data revealed that ECOG-PS of 2 was significantly associated with PFS, exhibiting an HR of 6.839 (95% CI: 1.557–30.032, p = 0.011) as shown in **Figure 6**.

### 3.4 Safety

Adverse events are summarized in **Table 4**. During the initial treatment with ICIs, the most common adverse events were fatigue (6 patients, 46.1%) and anemia (5 patients, 38.5%). Grade 3–4 events included anemia and neutropenia (2 patients each, 15.4%), leukopenia (1 patient, 7.8%), and fatigue (1 patient, 7.8%).In the ICIs rechallenge phase, anemia (5 patients, 38.5%) and fatigue (4 patients, 30.7%) remained the most frequently reported adverse events. However, only one Grade 3–4 event was observed, which was anemia (1 patient, 7.8%).

**Table 4:** Adverse events of ICIs initial treatment and ICIs re-challenge treatment.

Adverse events	Initial treatment of ICIs		Rechallenge of ICIs	
	Grade 1–2, n (%)	Grade 3–4, n (%)	Grade 1–2, n (%)	Grade 3–4, n (%)
Fatigue	6 (46.1)	1(7.8)	4 (30.7)	0
Decreased appetite	2 (15.4)	0	2 (15.4)	0
Diarrhea	1 (7.8)	0	1 (7.8)	0
Anemia	5 (38.5)	2 (15.4)	5 (38.5)	1 (7.8)
Neutropenia	3 (23.1)	2 (15.4)	1 (7.8)	0
Leukopenia	3 (23.1)	1 (7.8)	3 (23.1)	0
Hypothyroidism	0	0	1 (7.8)	0
Rash	2 (15.4)	0	1 (7.8)	0

## 4 Discussion

This study aims to evaluate the efficacy and safety of immune rechallenge therapy in the advanced NSCLC population with acquired immune resistance, based on Schoenfeld’s criteria. This is the first observational study to rigorously and comprehensively distinguish the population with acquired immune resistance. The results indicated that after immune rechallenge therapy, the median PFS of this population was 2.90 months (95% CI, 1.97-3.83), the DCR was 61.5%, and the toxicity was manageable. Further subgroup analysis revealed that the median PFS of the low PS group was significantly better than that of the high PS group (6.14 months *vs*. 1.68 months). Additionally, the results of univariate analysis indicated that a high PS score was an adverse prognostic factor for PFS. Our study demonstrates that, in real-world clinical practice, certain advanced patients with AIR can derive benefits from immune rechallenge therapy.

PIR and AIR represent the primary clinical challenges in further improving the prognosis of patients with advanced or metastatic lung cancer lacking driver genes. PIR is associated with the innate inability of the immune system to activate an appropriate immune response, which may arise from ineffective antigen presentation, T-cell priming, activation, trafficking, and migration, or even the baseline intratumoral overexpression of T-cell co-inhibitory receptors and immunosuppressive cells that the immunotherapeutic agent does not target (13). As for AIR, previous studies have demonstrated that the loss of key proteins involved in antigen presentation or defects in the IFNγ signaling pathway can contribute to immune resistance (14–16). A recent study systematically investigated the clinical and molecular features of AIR to immunotherapy in NSCLC (17). The findings revealed that AIR is closely associated with sustained activation of the IFN-γ signaling pathway, functional impairment of antigen presentation, T-cell exhaustion, and tumor-intrinsic unresponsiveness to IFN-γ stimulation, without the absence of immune cell infiltration. Specifically, the study revealed that in tumor cells exhibiting acquired resistance, there was an increase in the expression of IFNγ-induced genes, activation of STAT1 and IRF1, a CD8^+^ T cell exhaustion phenotype, aggregation of regulatory T cells (Tregs), mutations in antigen presentation-related molecules (such as β2 microglobulin), and a diminished response of tumor cells to sustained IFNγ stimulation. All these factors may contribute to the development of AIR. These mechanisms predominantly arise following the activation of the immune response, which starkly contrasts with the immune activation disorders observed in primary resistance, indicating substantial biological differences between the two resistance types. Although the mechanisms underlying AIR induced by PD-(L)1 blockers are not yet fully understood, the aforementioned research results provide an important preliminary basis for distinguishing the intrinsic mechanistic differences between PIR and AIR. Notably, different resistance patterns can significantly influence patients’ choices for subsequent treatment, particularly for those with AIR. In such cases, combining adjuvant therapies to reshape the immune microenvironment and reactivate the immune response may enable patients to benefit from immunotherapy once again.

As a potential option following the failure of first-line immunotherapy, immune rechallenge therapy has been validated by several studies in recent years regarding its efficacy and safety. Therefore, we systematically reviewed current studies on advanced NSCLC patients who underwent immunotherapy again after discontinuing initial ICIs treatment solely due to PD (**Table 5**). These studies are small-sample, retrospective, real-world studies, with sample sizes ranging from 12 to 165 cases (18–27). In terms of efficacy, the ORR ranged from 0% to 22.5%, the DCR ranged from 21.4% to 85%, the median PFS ranged from 1.6 to 6.8 months, and the median OS ranged from 6.5 to 14.8 months. The results of this study also fell within this range (median PFS: 2.9 months; ORR: 0%; DCR: 61.5%). Although the OS of this study is not yet mature, the median follow-up time is 10.31 months, suggesting that the median OS is expected to exceed this duration (**Appendix 1**). However, most of the studies above did not clearly distinguish between patients with AIR and those with PIR. Given the significant differences in immune resistance mechanisms and the small sample sizes, this methodological shortcoming could substantially affect the evaluation of the efficacy of immune rechallenge. Notably, the inclusion criteria established by Manyi Xu et al. are similar to those of this study; however, they do not specify a requirement for PD within six months following the last treatment with ICIs (26). Consequently, some patients who progress due to a prolonged drug withdrawal period may have been included in their analysis. In such instances, it becomes challenging to determine whether the progression is attributable to AIR or natural tumor progression resulting from the ineffectiveness of immune therapies. This is particularly relevant as most anti-PD-(L)1 antibodies maintain receptor occupancy in the body and activate endogenous anti-tumor immune responses for only a few months (28). Despite these limitations, the current research findings still indicate that immune rechallenge may confer certain clinical benefits to some patients with advanced NSCLC following first-line treatment with PD.

**Table 5:** Summary of ICIs rechallenge in retrospective real-world studies of patients who discontinued initial IO due to disease progression.

Author (year)	Sample size	Rechallenge of ICIs	ORR	DCR	mPFS	mOS	≥ grade 3 AEs
Kohei Fujita (2018) (18)	12	Nivo	8.3%	41.6%	3.1	NR	None
Hiromi Watanabe (2019) (19)	14	Nivo; Pembro	7.1%	21.4%	1.6	6.5	None
Yuki Katayama (2019) (20)	35	Nivo; Pembro; Atezo	2.9%	43%	2.7	7.5	NR
Kohei Fujita (2020) (21)	15	Nivo; Pembro	0	Nivo: 6.7% Pembro: 20.1%	Nivo: 1.9 Pembro:2.9	NR	interstitial pneumonia:6.7%; bacterial pneumonia :6.7%
Ziyi Xu (2022) (22)	40	NR	22.5%	85%	6.8	NR	NR
Masahiro Torasawa (2023) (25)	30	NR	6.7%	23.4%	2.2	NR	NR
Xiaoqi Yan (2024) (23)	165	NR	10.3%	66.7%	5.33	NR	15.2%
Jia Feng (2024) (24)	111	NR	17.1%	72.1%	5.9	14.3	18.0%
Manyi Xu (2024) (26)	104	NR	12.5%	76%	4.5	14.8	NR
Aram A Musaelyan (2024) (27)	52	Nivo; Pembro; Atezo	5.8%	NR	5.1	12.9	NR

mPFS, median progression-free survival; NR, not reported; mOS, median overall survival; Pembro, pembrolizumab; Nivo, Nivolumab; Atezo, Atezolizumab.

There is currently no consensus on which patients may benefit from rechallenge with immunotherapy. We propose that distinguishing between PIR and AIR is the first step in identifying the characteristics of potential beneficiaries of the rechallenge strategy. Commonly used markers of immune efficacy include PD-L1 expression and tumor mutation burden (29). However, in clinical practice, PD-L1 expression is rarely re-evaluated before retreatment. Our results indicated that the median PFS of PD-1-positive patients was superior to that of PD-1-negative patients (3.06 *vs*. 1.68 months). Although this difference was not statistically significant, it may have been influenced by the sample size. Previous studies have suggested that patients with an ECOG PS of 0-1, a neutrophil-to-lymphocyte ratio (NLR) of less than 3.8, and an objective response during initial ICIs treatment may benefit from immune rechallenge therapy, while those with a low body mass index (BMI ≤ 20) show no response to ICIs rechallenge (20, 27, 30). Similar findings were observed in our study, where the median PFS of the ECOG PS 0–1 group was greater than that of the ECOG PS 2 group (6.14 *vs*. 1.68 months). Further studies are necessary to confirm the potential influencing factors. Additionally, the construction of prognostic models that integrate single-cell data analysis and machine learning may yield valuable insights into predicting clinical outcomes for advanced NSCLC patients undergoing immune rechallenge, ultimately optimizing personalized treatment strategies.

During the immune rechallenge phase, the incidence of adverse events was generally lower than that observed during the initial treatment. Only one patient (7.8%) developed grade 3–4 anemia, while the remaining adverse events were classified as grade 1-2. Notably, no new serious immune-related adverse events occurred, indicating that the immune rechallenge was well tolerated.

This study has several limitations. First, this retrospective study involved a small sample size and was conducted at a single center, which precluded the multivariate analysis of confounding variables. The small sample size (n=13) limits the generalizability of our findings. Second, the rechallenge of ICIs was based on the physician’s judgment, resulting in potential selection bias. Third, there was a lack of assessment of PD-L1 expression before the initiation of ICIs rechallenge. Furthermore, biomarker data such as PD-L1 reassessment, tumor mutational burden (TMB), neutrophil-to-lymphocyte ratio (NLR), circulating tumor DNA (ctDNA), and tumor-infiltrating lymphocyte (TIL) density were not available, which limits the mechanistic interpretation of immune resistance in this study. Therefore, prospective validation in larger cohorts is warranted to confirm these findings.

## 5 Conclusions

Patients with advanced immune-acquired resistance non-small cell lung cancer who have failed first-line immunotherapy and lack driver gene mutations, particularly those with a good ECOG PS, may still benefit from immune rechallenge therapy. Our study, which adopts the newly defined AIR criteria, may help refine patient selection strategies and complement ongoing clinical trials on ICI rechallenge. Future large-scale prospective studies and molecular investigations are warranted to validate these findings and identify predictive markers to optimize rechallenge efficacy.

## Data Availability

The raw data supporting the conclusions of this article will be made available by the authors, without undue reservation.
