# Supplementary material for: Efficacy and safety of immune checkpoint inhibitor rechallenge therapy in the treatment of advanced acquired immune-resistant non-small cell lung cancer: a retrospective study
Source: Front Oncol. 2025 Sep 22;15:1621860. doi: 10.3389/fonc.2025.1621860 (PMC12497850; doi:10.3389/fonc.2025.1621860)
Supplement: Supplementary file 1 [file DataSheet1.docx]

**The clinical course of the patients treated with initial immunotherapy and ICI rechallenge therapy.**

| **Patient number** | **Histopathology** | **PDL-1 expression (%)** | **Initial treatment of ICIs** | | | **Interim treatments between initial and rechallenge ICI therapy** | **Time to rechallenge (months)** | **Rechallenge of ICIs** | | | |
| --- | --- | --- | --- | --- | --- | --- | --- | --- | --- | --- | --- |
|  |  |  | **Type of ICIs** | **Best Response** | **PFS**  **(months)** |  |  | **Type of ICIs** | **Best Response** | **PFS**  **(months)** | **OS**  **(months)** |
| Case 1 | Sq | <1 | TORI | PR | 12.09 | Yes | 9.30 | Tis | PD | 1.61 | 8.58 |
| Case 2 | Ad | 32 | Tis | PR | 6.97 | Yes | 4.53 | Cam | SD | 2.27 | 19.18 |
| Case 3 | Ad | 40 | Cam | PR | 2.40 | Yes | 21.30 | Sint | SD | 15.54 | 17.77 |
| Case 4 | Sq | <1 | Pembro | PR | 4.70 | Yes | 24.43 | Bema | PD | 1.41 | 4.04 |
| Case 5 | Sq | 23 | Pembro | PR | 4.50 | No | 3.25 | Pembro | SD | 2.83 | 12.48 |
| Case 6 | Ad | <1 | Tis | PR | 5.97 | Yes | 7.85 | Tis | PD | 1.68 | 5.26 |
| Case 7 | Ad | 45 | Pembro | PR | 9.67 | Yes | 1.41 | Pembro | SD | 6.14 | 18.92 |
| Case 8 | Ad | 20 | Pembro | PR | 5.13 | Yes | 4.00 | Pembro | SD | 2.90 | 3.87 |
| Case 9 | Ad | <1 | Tis | PR | 6.70 | Yes | 12.95 | Pembro | SD | 9.90 | 12.05 |
| Case 10 | AdSq | 42 | TORI | PR | 8.37 | Yes | 5.85 | Sintilimab | SD | 3.09 | 9.08 |
| Case 11 | Sq | 90 | Tis | PR | 5.33 | Yes | 9.23 | Tis | PD | 3.06 | 7.01 |
| Case 12 | Sq | 26 | Tis | PR | 19.27 | No | 3.33 | Tis | SD | 4.37 | 13.23 |
| Case 13 | Sq | <1 | Tis | PR | 3.00 | Yes | 7.53 | Sint | PD | 2.20 | 10.31 |

Ad, adenocarcinoma; Sq, squamous cell carcinoma; AdSq, Adenosquamous carcinoma; ICIs: immune checkpoint inhibitors; PFS, progression-free survival; PR, partial response; SD, stable disease; PD, progression disease; TORI, Toripalimab; Pembro, pembrolizumab; Tis, Tislelizumab; Cam, Camrelizumab; Sint, Sintilimab; Bema, Bemarituzumab;
