# Supplementary material for: Efficacy and safety of immune checkpoint inhibitor rechallenge therapy in the treatment of advanced acquired immune-resistant non-small cell lung cancer: a retrospective study
Source: Front Oncol. 2025 Sep 22;15:1621860. doi: 10.3389/fonc.2025.1621860 (PMC12497850; doi:10.3389/fonc.2025.1621860)
Supplement: Supplementary file 2 [file DataSheet2.docx]

Summary of initial immunotherapy and immune checkpoint inhibitor rechallenge for each patient.

| Initial immunotherapy regimen | Immune re-challenge therapy |
| --- | --- |
| Tislelizumab + Albumin-bound paclitaxel | Tislelizumab |
| Camrelizumab + Carboplatin + Pemetrexed | Sintilimab + Anlotinib |
| Sintilimab + Pemetrexed + Carboplatin | Sintilimab |
| Toripalimab + Albumin-bound paclitaxel + Cisplatin | Benmelstobart + Anlotinib |
| Toripalimab + Albumin-bound paclitaxel + Nedaplatin | Toripalimab |
| Camrelizumab + Paclitaxel + Cisplatin | Docetaxel + Tislelizumab |
| Toripalimab + Albumin-bound paclitaxel + Carboplatin + Bevacizumab | Toripalimab + Bevacizumab |
| Toripalimab + Pemetrexed + Cisplatin + Bevacizumab | Toripalimab |
| Camrelizumab + Carboplatin + Pemetrexed | Toripalimab |
| Tislelizumab + Albumin-bound paclitaxel + Nedaplatin | Sintilimab + Gemcitabine + Nedaplatin |
| Camrelizumab + Paclitaxel + Cisplatin | Tislelizumab + Albumin-bound paclitaxel |
| Camrelizumab + Paclitaxel + Nedaplatin | Tislelizumab + Albumin-bound paclitaxel |
| Camrelizumab + Paclitaxel + Cisplatin | Sintilimab |
